# Exploring intensivist involvement: Patient characteristics, interventions and outcomes

**DOI:** 10.5578/tt.202402940

**Published:** 2024-06-12

**Authors:** Hüseyin ARIKAN, Ayvaz YELER, Ramazan ESEN

**Affiliations:** 1 Unit of Internal Medicine Intensive Care, Van Yüzüncü Yıl University, Dursun Odabaş Medical Center, Van, Türkiye; 2 Department of Internal Medicine, Van Yüzüncü Yıl University Faculty of Medicine, Van, Türkiye

## Abstract

**ABSTRACT**

**
Exploring intensivist involvement: Patient characteristics, interventions and outcomes
**

**Introduction:** *Intensivists play a critical role in the management of intensive care units (ICUs) and in providing high quality care. While international guidelines recommend intensivist staffing for improved patient outcomes, there is a shortage of qualified intensivists in many regions, including Türkiye. This study aimed to assess the impact of introducing a full-time intensivist to a medical ICU on patient characteristics, outcomes, and ICU interventions.
*

**Materials and Methods:** *This retrospective study analyzed data from the Internal Medicine ICU at Van Yüzüncü Yıl University Dursun Odabaş Medical Center over two periods: Pre- and post-intensivist recruitment. The study included adult patients admitted to the ICU from February 2018 to January 2020. Patient demographics, reasons for ICU admission, APACHE-II and SOFA scores, ICU interventions, and outcomes were recorded and compared between the two periods.
*

**Results:** *Of the 868 patients admitted during the study period, 820 were included in the analysis. There were no significant differences in demographic characteristics between the pre- and post-intensivist periods. However, patients in the post-intensivist period had higher APACHE-II and SOFA scores. Intensive care units mortality rates were comparable between the two peri- ods. The post-intensivist period saw increased use of invasive mechanical ventilation and non-invasive ventilation compared to the pre-intensivist peri- od. Renal replacement therapy usage and enteral nutrition provision also increased in the post-intensivist period. ICU and hospital lengths of stay remained similar between the two periods.
*

**Conclusion:** *The introduction of a full-time intensivist to the medical ICU led to changes in ICU interventions, including increased use of mechanical venti- lation and renal replacement therapy. Despite these changes, ICU mortality rates remained unchanged. Further research is needed to explore the long- term impact of intensivist staffing on patient outcomes in Türkiye.
*

**Key words:** *Intensivist; mortality; intensive care
*

**ÖZ**

**
Yoğun bakım uzmanının katılımının incelenmesi: Hasta özellikleri, müdahaleler ve sonuçlar
**

**Giriş:** *Yoğun bakım uzmanları, yoğun bakım ünitelerinin (YBÜ) yönetiminde ve yüksek kaliteli bakım sağlamada kritik bir rol oyna- maktadır. Uluslararası kılavuzlar, hasta sonuçlarını iyileştirmek için yoğun bakım uzmanlarının görevlendirilmesini önerirken, birçok bölgede, özellikle Türkiye’de nitelikli yoğun bakım uzmanı eksikliği bulunmaktadır. Bu çalışma, bir YBÜ’ye tam zamanlı bir yoğun bakım uzmanının atanmasının hasta özellikleri, sonuçları ve YBÜ müdahaleleri üzerindeki etkisini değerlendirmeyi amaçlamaktadır.
*

**Materyal ve Metod:** *Van Yüzüncü Yıl Üniversitesi Dursun Odabaş Tıp merkezi İç Hastalıkları YBÜ’deki verileri yoğun bakım uzmanı- nın göreve başlamasından önceki ve sonraki iki dönemde analiz etmiştir. Çalışmaya, Şubat 2018-Ocak 2020 tarihleri arasında YBÜ’ye kabul edilen yetişkin hastalar dahil edilmiştir. Hasta demografileri, YBÜ’ye kabul nedenleri, APACHE-II ve SOFA skorları, YBÜ müda- haleleri ve sonuçları kaydedilmiş ve iki dönem arasında karşılaştırılmıştır.
*

**Bulgular:** *Çalışma döneminde kabul edilen 868 hastadan 820’si analize dahil edilmiştir. Öncesi ve sonrası dönemlerde demografik özellikler arasında önemli bir fark bulunmamıştır. Ancak, sonrası dönemdeki hastaların APACHE-II ve SOFA skorları daha yüksek bulunmuştur. Yoğun bakım üniteleri mortalite oranları iki dönem arasında benzerdir. Sonrası dönemde invaziv mekanik ventilasyon ve non-invaziv ventilasyon kullanımı artmıştır. Renal replasman tedavisi kullanımı ve enteral beslenme sağlanması da sonrası dönem- de artmıştır. Yoğun bakım üniteleri ve hastane yatış süreleri iki dönem arasında benzer kalmıştır.
*

**Sonuç:** *Dahili YBÜ’ye tam zamanlı bir yoğun bakım uzmanının atanması, mekanik ventilasyon ve renal replasman tedavisinin artma- sı da dahil olmak üzere YBÜ müdahalelerinde değişikliklere yol açmıştır. Bu değişikliklere rağmen, YBÜ mortalite oranları değişme- miştir. Türkiye’de yoğun bakım uzmanlarının görevlendirilmesinin hasta sonuçları üzerindeki uzun vadeli etkisini araştırmak için daha fazla çalışmaya ihtiyaç vardır.
*

**Anahtar kelimeler:** *Yoğun bakım uzmanı; mortalite; yoğun bakım
*

## INTRODUCTION

Intensivists are crucial for the effective management of intensive care units (ICUs) and for providing high- quality intensive care. Staffing ICUs with intensivists enhances the quality of care and leads to improved clinical outcomes, including reduced mortality rates, shorter durations of mechanical ventilation, and decreased ICU length of stay (LOS) (1,2) . Both the Society of Critical Care Medicine’s guidelines for ICU admission, discharge, and triage in the United States and the Leapfrog standards for critical care recom- mend that ICUs be staffed by intensivists who can coordinate and oversee the care of critically ill patients (3,4). Additionally, ICU facility standards mandate the presence of a doctor whose primary focus is working in the ICU.

Despite the widely acknowledged importance of intensivists, there are not enough qualified intensivists to staff all ICUs in Türkiye (5,6). Typically, in our country, physicians from the anesthesiology and reanimation clinics oversee patient care (7). Specialization training in intensive care medicine commenced in 2012, with the first multidisciplinary intensivists assuming roles in 2016. However, there remains insufficient data regarding the impact of intensivists on patient outcomes in multidisciplinary ICUs in Türkiye.

In this study, we aimed to investigate the effect of an intensivist who started working in a medical ICU on

patients’ characteristics, outcomes and ICU interventions.

### MATERIALS and METHODS

**Study Design**

This retrospective study evaluates the performance of the Internal Medicine ICU at Van Yüzüncü Yıl University Dursun Odabaş Medical Center, which comprises 15 beds, during two distinct time periods. A dedicated full-time intensivist joined the team in February 2019. Data from the preceding year (February 2018-January 2019) and the subsequent year (February 2019-January 2020) were analyzed. Institutional review board approval was obtained for this study from Van Yüzüncü Yıl University Non- Interventional Clinical Research Ethics Committee (Decision date: 07.02.2020, Decision no: 2020/02- 12). Given its retrospective nature, the requirement for informed consent was waived by the IRB.

### Settings

The Internal Medicine Intensive Care Unit at Van Yüzüncü Yıl University Dursun Odabaş Medical Center is equipped with 15 beds and is certified as a level III ICU. It offers invasive monitoring, invasive and non-invasive mechanical ventilation, and bedside hemodialysis services. The nursing staff remained consistent across the periods under review, with a staffing ratio of one nurse per two patients. Prior to February 2019, daytime physicians worked in monthly

rotations alongside two research assistants from the department of internal medicine. Each specialty and subspecialty admitting patients to the ICU conducted their own rounds, following a low-intensity model. In February 2019, the introduction of an intensivist to the ICU team marked a shift to a high-intensity model. The intensivist, supported by specialists and two research assistants from the internal medicine department, assumed leadership. Night shift and holiday schedules remained unchanged, with a permanent ICU staff member present. The intensivist was available on-call via telephone during holidays and nights when not physically present. Daily multidisciplinary rounds were conducted, with the intensivist overseeing ICU operations.

### Patients

Throughout the study period, we evaluated adult patients (aged ≥18 years) admitted to the ICU for potential inclusion. Patients with ICU stays lasting less than 24 hours, those who passed away within the initial 24 hours of admission, and individuals admitted solely for postoperative care were excluded from the analysis. Additionally, repeated ICU admissions-instances where patients were discharged from and subsequently readmitted to the ICU during the same hospital stay-were omitted from consideration. Included patients were classified into two groups: Those receiving low-intensity care and those receiving high-intensity care.

### Data Collection

The participants’ data included age, sex, reason for ICU admission, length of ICU stay, APACHE-II scores, Charlson comorbidity index (CCI), requirement for mechanical ventilation (MV), need for renal replacement therapy (RRT), need for vasopressors,

and in-hospital mortality during ICU admission. Patient admission reasons were categorized as sepsis, respiratory issues, cardiac conditions, neurological disorders, post-cardiopulmonary arrest, and other causes. The use of MV and, if applicable, whether it was invasive or non-invasive (NIV), was noted during ICU admission.

### Outcome Assessment

The primary outcome was defined as mortality within the ICU. Secondary outcomes encompassed interventions within the ICU, such as invasive or non- invasive MV, renal replacement therapies, nutritional support, ICU and hospital length of stay (LoS).

### Statistical Analysis

Statistical analysis utilized the Statistical Package for the Social Sciences (SPSS, Version 22.0, Chicago, IL). Bivariate analyses involved the chi-square test for categorical variables, the t-test for normally distributed continuous variables, and the Wilcoxon test for non-normally distributed continuous variables. Descriptive statistics for quantitative variables were presented as mean with standard deviation or median with interquartile range, while qualitative variables were expressed as frequency and percentage. The significance level was set at 0.05.

## RESULTS

### Baseline Characteristics

Throughout the study period, a total of 868 patients were admitted to the internal medicine ICU. Of these, 376 were admitted during the low-intensity care period, and 492 during the high-intensity care period. For analysis, 351 patients from the low- intensity period and 469 from the high-intensity

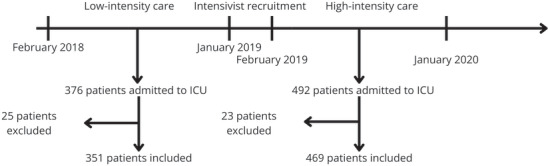

**Figure 1.** Flow chart.

**Table:** 

**Table 1.** Characteristics of participants during the low-intensity versus high-intensity era			
**Characteristic**	**Low-Intensity ICU Era (n= 351)**	**High-Intensity ICU Era (n= 469)**	**p**
Age	68 (52-83)	67 (55-81)	0.60
Sex, male	210 (59.8)	298 (53.5)	0.28
CCI	1 (0-2)	1 (0-2)	0.78
Reason for admission			
Sepsis	50 (14.3)	70 (14.9)	
Respiratory issues	126 (35.9)	183 (39)	0.96
Cardiac conditions	105 (29.9)	136 (28.9)	
Other	70 (19.9)	80 (17.2)	
Admitted from			
Emergency service	106 (30.2)	141 (30.1)	
			0.98
Wards	210 (59.8)	281 (59.9)	
Another hospital	35 (10)	47 (10)	
APACHE II	24 (19-31)	26 (22-41)	0.048
SOFA	4 (2-8)	6 (3-10)	0.045
CCI: Charlson comorbidity index, APACHE II: Acute physiology and chronic health evaluation II, SOFA: Sepsis-related organ failue assessment; Continuous variables are represented as median (interquartile range), categoric variables represented as n (%).

period were included (Figure 1). There were no significant differences in demographic characteristics, such as age, sex, reason for ICU admission, and CCI, between the two groups. However, patients admitted during the high-intensity care period had notably higher APACHE-II and SOFA scores (Table 1).

### Primary Outcome

Intensive care units mortality was 38.5% with 135 cases in the low intensity care group, whereas it was 42.4% with 199 cases in the high intensity care group (p= 0.25). Compared to the low-intensity ICU era, the high-intensity ICU era was associated with an increased mortality in unadjusted analysis (OR= 1.18; 95% CI= 0.89 to 1.56; p= 0.13).

### Secondary Outcomes

Invasive MV was performed in 133 (37.9%) patients during low-intensity ICU era; however, it was performed in 244 (52%) patients during high-intensity ICU era (p< 0.001). Interestingly, there was no instance of NIV in the low-intensity group. In the high-intensity group, 34 (7.2%) patients received non- invasive mechanical ventilation support (p< 0.001).

Renal replacement therapy was available as bed side conventional hemodialysis. At the low-intensity care group, 42 (11.9%) patients were supported by RRT, whereas at the high intensity group 68 (14.5%) patients required RRT (p= 0.29).

Nutritional support via enteral route was evaluated in intubated patients. In the low-intensity era, none of the intubated patients had enteral nutrition. All intubated patients were given parenteral nutrition. However, in the high-intensity era, nearly all intubated patients were provided enteral nutrition. Of the 244 intubated patients, only 38 patients were not provided enteral nutrition due to gastrointestinal obstruction caused by malignancies.

There was no clear impact of ICU structure on length of stay. Hospital stay was similar in the low- and high-intensity ICU eras (median 23 vs. 27 days, respectively; p= 0.62). ICU length of stay was also similar for the low- and high-intensity ICU eras (12 vs. 10 days, p= 0.17).

## DISCUSSION

In this current study, we found that the introduction of a full-time intensivist to the medical ICU led to changes in ICU interventions, including increased use of mechanical ventilation and renal replacement therapy. Despite these changes, we found that ICU mortality rates remained unchanged.

When ICU staffing transitions from low-intensity to high-intensity, significant improvements in outcomes are observed in various ICU settings (1,2,8-9). The Leapfrog Group’s ICU Physician Staffing Safety Standards advocate for high-intensity staffing based

on previous studies (4). However, some research has shown that ICU management does not always improve mortality rates and its effectiveness remains debated, despite some guidelines recommending the placement of intensivists in the ICU (3,4,8-11). It is intuitive to think that the absence of intensivists might reduce the quality of ICU management. Nonetheless, our study found that shifting from low- intensity to high-intensity ICU staffing did not significantly affect clinical outcomes for all-cause mortality. The impact of intensivists on mortality might not be as significant as earlier studies have suggested (12). Earlier studies indicating a relationship between mortality reduction and intensivist staffing were mostly from single-center, before-and-after analyses conducted in the 1990s or 2000s (1,2). Moreover, ICU and hospital mortality rates have varied by decade, decreasing in the 1980s and 2000s but not in the 1990s or 2010s (2). Recent multicenter analyses have shown no significant association between high-intensity ICU staffing and mortality (13,14). These findings suggest that the relationship between intensivist staffing and patient mortality is weaker than previously thought. The transition to high-intensity staffing likely has only a limited impact on mortality in small- and medium-volume hospitals. A closed ICU with a 24/7 intensivist staffing model in an academic hospital reduced the LoS and generated significant cost savings (15). Additionally, a recent study has revealed that 24/7 in-house ICU intensivists positively affect the quality of care for critically ill patients in high-acuity, high-volume centers; however, these benefits are not sufficiently applicable to low-acuity, low-volume hospitals to justify the increased staffing needs and costs (16).

The observed increase in invasive MV from 37.9% during the low-intensity ICU era to 52% in the high- intensity ICU era underscores the impact of intensivist presence on clinical decision-making. High-intensity ICU settings, characterized by the constant availability of intensivists, likely facilitate more aggressive management strategies, including the timely initiation of invasive MV. This availability may result in admitting more severe patients and aligns with the existing literature, which suggests that intensivist-led care improves the coordination and execution of complex interventions (1,2,17,18). Both in the recent studies by Baik et al. and Ko et al., after implementation of a high intensity care protocol, frequency of invasive MV increased. However, this increase did

not reach statistical significance.

The absence of NIV in the low-intensity group and its utilization in 7.2% of the patients in the high- intensity group highlight the evolution of respiratory support practices. High-intensity ICUs are better equipped to implement NIV due to the continuous presence of trained staff who can manage potential complications and adjust treatment plans swiftly. However, this was not the case in our unit. When the intensivist first joined the team, ICU did not have necessary equipment to implement NIV. A few months later, necessary equipment was provided. Thus, current ratios may be misleading because there were missed opportunities. But this shift reflects a broader acceptance of NIV in high-intensity settings, supported by evidence of its benefits in appropriately selected patients (19).

The use of RRT showed a non-significant increase from 11.9% in the low-intensity group to 14.5% in the high-intensity group. This lack of significant difference suggests that the decision to initiate RRT is primarily driven by patient-specific factors rather than the staffing model. The literature indicates that RRT is typically initiated based on clinical criteria, such as the severity of acute kidney injury (AKI) and overall patient stability, which may not be directly influenced by the presence of intensivists (20). However, this should be noted that continuous renal replacement therapies were not available in this study setting. The volatile nature of critical care patients is obvious thus conventional hemodialysis is not a first line choice in critical care settings. A certain number of patients with RRT indication could not have it because of hemodynamic instability.

A significant shift was observed in the method of nutritional support for intubated patients. In the low- intensity era, all intubated patients received parenteral nutrition, while in the high-intensity era, nearly all intubated patients were provided enteral nutrition. This change is indicative of the growing preference for enteral nutrition, which is associated with improved outcomes, such as lower infection rates and better gastrointestinal function (21). The ability to provide enteral nutrition in high-intensity settings reflects the enhanced capability of these ICUs to implement best practices. High-intensity ICUs likely have better resources and more specialized staff to manage enteral feeding, including addressing complications and ensuring optimal nutrient delivery.

This shift is supported by guidelines recommending enteral over parenteral nutrition for critically ill patients due to its physiological benefits and lower risk of complications (21).

Our study found no significant difference in hospital or ICU length of stay between the low- and high- intensity ICU eras. This finding aligns with some previous studies, which suggest that high-intensity staffing does not necessarily reduce length of stay (8). The complexity and severity of illnesses treated may play a more substantial role in determining length of stay than the staffing model alone. Moreover, factors such as discharge planning, availability of post-ICU care facilities, and patient recovery rates also influence length of stay (22).

## CONCLUSION

In conclusion, our study highlights significant differences in mechanical ventilation practices, nutritional support, and the limited impact on RRT utilization and length of stay between low- and high- intensity ICU eras. These findings suggest that while high-intensity ICU staffing enhances the quality of certain interventions, its overall impact on patient outcomes, particularly length of stay, may be less pronounced. A comprehensive approach that integrates staffing models with other clinical practices and resource optimization is essential for improving ICU care.

**Ethical Committee Approval:** This study was approved by Van Yüzüncü Yıl University Non-Interventional Clinical Research Ethics Committee (Decision Date: 07.02.2020, Decision No: 2020/02-12).

### CONFLICT of INTEREST

The authors declare that they have no conflict of interest.

## AUTHORSHIP CONTRIBUTIONS

Concept/Design: HA, RE Analys/Interpretation: HA, AY Data acqusition: AY

Writing: HA, AY

Clinical Revision: HA, RE Final Approval: HA, AY, RE

## REFERENCES

1. *
  Pronovost PJ, Angus DC, Dorman T, Robinson KA, Dremsizov TT, Young TL. Physician staffing patterns and clinical outcomes in critically ill patients: A systematic review. Jama 2002; 288(17): 2151-62. https://doi.org/10.1001/jama.288.17.2151*
2. *
  Wilcox ME, Chong CA, Niven DJ, Rubenfeld GD, Rowan KM, Wunsch H, et al. Do intensivist staffing patterns influence hospital mortality following ICU admission? A systematic review and meta-analyses. Crit Care Med 2013; 41(10): 2253-74. https://doi.org/10.1097/CCM.0b013e318292313a*
3. *
  Nates JL, Nunnally M, Kleinpell R, Blosser S, Goldner J, Birriel B, et al. ICU admission, discharge, and triage guide- lines: A framework to enhance clinical operations, devel- opment of institutional policies, and further research. Crit Care Med 2016; 44(8): 1553-602. https://doi.org/10.1097/CCM.0000000000001856*
4. *
  Manthous CA. Leapfrog and critical care: Evidence- and reality-based intensive care for the 21st century. Am J Med 2004; 116(3): 188-93. https://doi.org/10.1016/j.amjmed.2003.08.032*
5. *
  Güneş M, Şenoğlu N. Employees and staff models in inten- sive care unit. In: Hancı V, (ed). Organization, Management and Training in Intensive Care. 1ed. Türkiye: Türkiye Klinikleri, 2020:18-25.
  *
6. *
  Arıkan H, Ürkmez S, Yardaş G, Başar H, Turan I, Demirkıran O. A current overview of intensive care sub- speciality education in Turkey: What do educational staff, subspecialty residents and specialists think? Turk J Intensive Care 2023; 21(4): 288-96. https://doi.org/10.4274/tybd.galenos.2023.78941*
7. *
  Ediboğlu Ö, Moçin ÖY, Özyılmaz E, Saltürk C, Önalan T, Seydaoğlu G, et al. Current statement of intensive care units in Turkey: Data obtained from 67 Centers. Turk Thorac J 2018; 19(4): 209-15. https://doi.org/10.5152/TurkThoracJ.2018.170104*
8. *
  Kim DJ, Sohn B, Kim H, Chang HW, Lee JH, Kim JS, et al. The impact of an attending intensivist on the clinical out- comes of patients admitted to the cardiac surgical inten- sive care unit after coronary artery bypass grafting. Korean J Thorac Cardiovasc Surg 2020; 53(1): 8-15. https://doi.org/10.5090/kjtcs.2020.53.1.8*
9. *
  Miller PE, Chouairi F, Thomas A, Kunitomo Y, Aslam F, Canavan ME, et al. Transition from an open to closed staffing model in the cardiac intensive care unit improves clinical outcomes. J Am Heart Assoc 2021; 10(3): e018182. https://doi.org/10.1161/JAHA.120.018182*
10. *
  Nagendran M, Dimick JB, Gonzalez AA, Birkmeyer JD, Ghaferi AA. Mortality among older adults before versus after hospital transition to intensivist staffing. Med Care 2016; 54(1): 67-73. https://doi.org/10.1097/MLR.0000000000000446*
11. *
  Wise KR, Akopov VA, Williams BR Jr, Ido MS, Leeper KV Jr, Dressler DD. Hospitalists and intensivists in the medical ICU: A prospective observational study comparing mor- tality and length of stay between two staffing models. J Hosp Med 2012; 7(3): 183-9. https://doi.org/10.1002/jhm.972*
12. *
  Costa DK, Wallace DJ, Kahn JM. The association between daytime intensivist physician staffing and mortality in the context of other ICU organizational practices: A multi- center cohort study. Crit Care Med 2015; 43(11): 2275-
  *

*
82. https://doi.org/10.1097/CCM.0000000000001259*

13. *
  Checkley W, Martin GS, Brown SM, Chang SY, Dabbagh O, Fremont RD, et al. Structure, process, and annual ICU mortality across 69 centers: United States critical illness and injury trials group critical illness outcomes study. Crit Care Med 2014; 42(2): 344-56. https://doi.org/10.1097/CCM.0b013e3182a275d7*
14. *
  Yoo EJ, Edwards JD, Dean ML, Dudley RA. Multidisciplinary critical care and intensivist staffing: Results of a statewide survey and association with mortality. J Intensive Care Med 2016; 31(5): 325-32. https://doi.org/10.1177/0885066614534605*
15. *
  Banerjee R, Naessens JM, Seferian EG, Gajic O, Moriarty JP, Johnson MG, et al. Economic implications of night time attending intensivist coverage in a medical intensive care unit. Crit Care Med 2011; 39(6): 1257-62. https://doi.org/10.1097/CCM.0b013e31820ee1df*
16. *
  Masud F, Lam TYC, Fatima S. Is 24/7 in-house intensivist staffing necessary in the intensive care unit? Methodist Debakey Cardiovasc J 2018; 14(2): 134-40. https://doi.org/10.14797/mdcj-14-2-134*
17. *
  Ko SJ, Cho J, Choi SM, Park YS, Lee CH, Yoo CG, et al. Impact of staffing model conversion from a mandatory critical care consultation model to a closed unit model in the medical intensive care unit. PLoS One 2021; 16(10): e0259092. https://doi.org/10.1371/journal.pone.0259092*
18. *
  Baik SM, Yang NR, Kim TY, Hong KS. Excellence of the closed intensive care unit system in critically ill surgical patients. PLoS One 2023; 18(4): e0285035. https://doi.org/10.1371/journal.pone.0285035*
19. *
  Rochwerg B, Brochard L, Elliott MW, Hess D, Hill NS, Nava S, et al. Official ERS/ATS clinical practice guidelines: Non-invasive ventilation for acute respiratory failure. Eur Respir J 2017; 50(2): 1602426. https://doi.org/10.1183/13993003.02426-2016*
20. *
  Mehta RL, Cerdá J, Burdmann EA, Tonelli M, García- García G, Jha V, et al. International Society of Nephrology’s 0by25 initiative for acute kidney injury (zero preventable deaths by 2025): A human rights case for nephrology. Lancet 2015; 385(9987): 2616-43. https://doi.org/10.1016/S0140-6736(15)60126-X*
21. *
  Taylor BE, McClave SA, Martindale RG, Warren MM, Johnson DR, Braunschweig C, et al. Guidelines for the provision and assessment of nutrition support therapy in the adult critically Ill patient: Society of Critical Care Medicine (SCCM) and American Society for parenteral and enteral nutrition (A.S.P.E.N.). Crit Care Med 2016; 44(2): 390-438. https://doi.org/10.1097/CCM.0000000000001525*
22. *
  Zilberberg MD, Luippold RS, Sulsky S, Shorr AF. Prolonged acute mechanical ventilation, hospital resource utilization, and mortality in the United States. Crit Care Med 2008; 36(3): 724-30. https://doi.org/10.1097/CCM.0B013E31816536F7*

